# The PRINCIPAL Network: A Model to Optimize Infection Care and Prevention in Pediatric Oncology in the Latin American Region

**DOI:** 10.1200/GO.22.00187

**Published:** 2022-11-29

**Authors:** Miguela A. Caniza, Maysam R. Homsi, Milka Vázquez, Ivan F. Gutierrez, Ioannis Kopsidas, Sheena Mukkada, Paola Friedrich, Monika Metzger

**Affiliations:** ^1^ Department of Global Pediatric Medicine, St Jude Children's Research Hospital, Memphis, TN; ^2^ Department of Infectious Diseases, St Jude Children's Research Hospital, Memphis, TN; ^3^ Department of Pediatrics, University of Tennessee Health Science Center, Memphis, TN; ^4^ Clínica Infantil Colsubsidio, Bogotá, CO; ^5^ 2nd Department of Pediatrics and Center for Clinical Epidemiology and Outcomes Research, National and Kapodistrian University of Athens, Athens, Greece; ^6^ Department of Oncology, St Jude Children's Research Hospital, Memphis, TN

## Abstract

Children with cancer are at high risk for poor outcomes, and health care providers are often unfamiliar with best practices in infection care and prevention (IC&P) in this small and fragile population. Graduates of training courses in IC&P in immunocompromised hosts identified a need for a community that would enable members to share health care experiences, provide resources for continuing medical education, and foster collaborative research and quality improvement opportunities. We developed a Latin American network, Prevencionistas e Infectólogos para Cáncer Pediátrico en América Latina, to grow and sustain the expertise of the clinical workforce in IC&P. Here, we describe the network, how we built it, and its early outcomes.

**METHODS:**

We began by codesigning the mission, vision, objectives, and values. We then established the structure for leadership and network management to provide a functional uniformity and sustainability. Virtual meetings with network members and strategic in-person gatherings optimized the use of the time and resources of the network.

**RESULTS:**

The network has seen good participation by members and candidates for membership, who have provided feedback on case-based learning. Members have attended training sessions on quality improvement, research in human subjects, and IC&P in pediatric oncology at national and regional meetings and workshops. Network members have presented their work at regional and global meetings, and publications are beginning to emerge from this community. A direct effect of the Prevencionistas e Infectólogos para Cáncer Pediátrico en América Latina network has been the creation of a similar network for the Asia Pacific region, and a third network is being planned.

**CONCLUSION:**

We have demonstrated the power of a discipline-specific network structure to facilitate sharing of evidence-based information that enhances the quality-of-care delivery in pediatric oncology.

## INTRODUCTION

The survival of children with cancer in low- and middle-income countries (LMICs) remains poor,^1^ whereas in high-income countries, it has surpassed 85%.^2^ This unequal outcome reflects the more advanced stage of disease at diagnosis, the higher rate of treatment abandonment, and the high treatment-related mortality, mainly because of infections, in LMICs.^3^ Outcomes of care for children in LMICs can be improved by implementing measures to prevent and/or control infections. Such measures to improve the quality of care rely on the presence of a sufficient number of health care providers with the necessary expertise.^4^

**CONTEXT**

**Key Objective**
The Prevencionistas e Infectólogos para Cáncer Pediátrico en América Latina (PRINCIPAL) network is a community of health care professionals focused on decreasing the risk for poor outcomes of infections in children in Latin America.
**Knowledge Generated**
The network stimulates knowledge exchange, cross-mentoring, and collaboration. Both intraregional collaboration and inter-regional collaboration allow for a unified approach to address mutual concerns and for advocacy to global agencies.
**Relevance**
PRINCIPAL is an imperative to improving infection-related care and has progressively evolved to its current structure. Although St Jude Global has supported its inception and early performance, PRINCIPAL is envisioned to become a self-sustaining structure with active participation of other institutions, organizations, and agencies to strengthen its work. A lasting effect of the network will be the improvement of local infectious disease expertise and management; and the sharing of relevant data that will lead to better patient outcomes.

In many LMICs, a shortage of skilled health care professionals hinders the provision of high-quality care, and postgraduate education and training are often unavailable.^5^ In such an environment, knowledge sharing, fluid communication, and collaboration between health care professionals can maximize the use of their limited time and expertise. Regional and global professional networks in infection care and prevention (IC&P) with focused goals and objectives can be resources for capacity building, education and studies in clinical research, quality improvement, and implementation science.^6-9^

**FIG 1:**
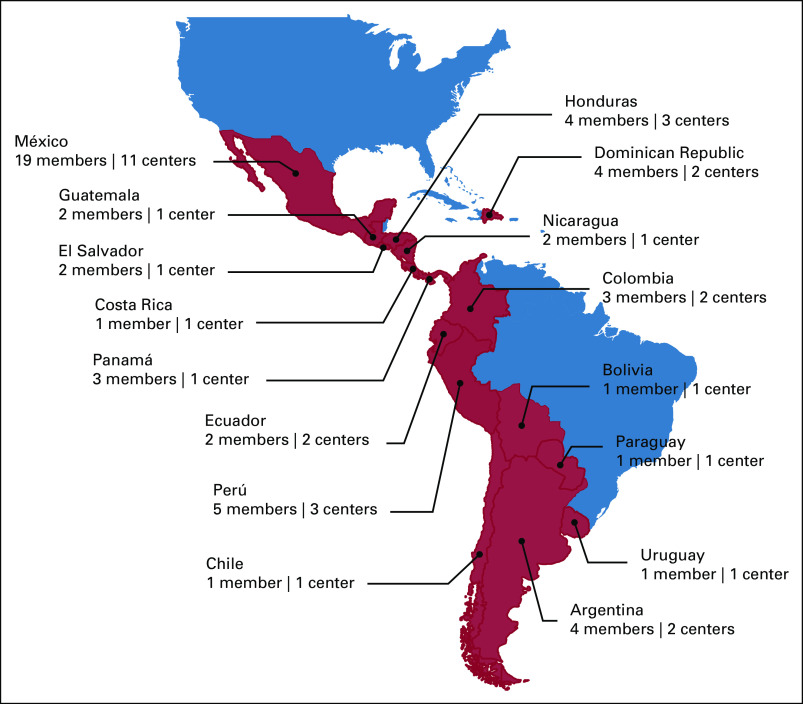
Prevencionistas e Infectólogos para Cáncer Pediátrico en América Latina Network representation since 2017.

In 2017, we initiated an educational program for health care providers caring for children with cancer and infections.^10^ The alumni of the first training cohort established a network that remained active after the completion of the course (Fig 1, Table 1). Here, we describe how this network was built, organized, and sustained. Additionally, we share how this type of professional network can be used to stimulate medical education and collaboration in IC&P and thereby serve as a model to improve the quality-of-care delivery for children with cancer.

**TABLE 1:**
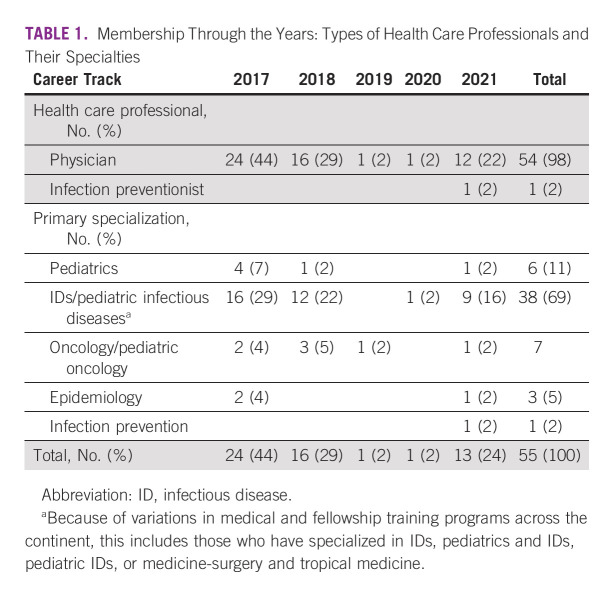
Membership Through the Years: Types of Health Care Professionals and Their Specialties

## METHODS

### Resources

#### The St Jude Global infectious disease program.

The goal of the Global infectious disease (Global ID) program^11^ is to help decrease the rate of and risks for infection among children with cancer, mainly—but not exclusively—at institutions that are members of the St Jude Children's Research Hospital (St Jude) Global Alliance. The administrative and programmatic financial support for Global ID program is provided by St Jude. Three coordinators dedicate up to 40% time to managing the networks as part of their responsibilities in the Global ID program. This program works to develop and promote standards for IC&P by establishing, participating in, and facilitating capacity-building initiatives, education and training opportunities, and research studies on infectious disease (ID)–related topics at collaborative sites around the world. To satisfy the learning needs of collaborators, the Global ID Program established two main training courses: the ID Training Seminars for leaders^10^ and the Intensive Infection Control Course for preventionists.^12^ The Global ID Program stimulates communication with and among participants during and after completion of the courses by inviting them to participate in program activities, such as contributing to a monthly bulletin or communicating via social media venues, for example, WhatsApp. As these engagements were not sufficient by themselves, and as the number of course graduates grew over time, the need for a more sustainable and more organized structure for engaging with Global ID and with each other was increasingly apparent. Accordingly, 2017 saw the birth of the concept of a network to address the needs of participants with respect to IC&P capacity building, education, and research.^10^

#### The St Jude Global ID training seminars for leaders.

This is a 10-week blended course in IC&P consisting of 8 weeks of distance learning followed by 2 weeks of in-person learning. Since its launch in 2017,^10^ 189 participants have attended this course. Overall, participants showed improved knowledge in topics taught and positive behavioral changes in their institutional engagement. The members of each cohort of participants bond throughout their training and continue to communicate and cooperate with each other and the Global ID Program after graduation, including joining and participating in our networks.

#### The St Jude Infection Prevention and Control course for preventionists.

This is a 10-week blended course in infection prevention and control (IPC) for infection preventionists. It consists of 9 weeks of distance learning followed by 1 week of in-person learning. The original version of the course, consisting of 4 weeks of in-person learning, was launched in 2005^12^ but subsequently underwent a series of transformations to accommodate the needs of the audience. The course is offered in Spanish and English and, to date, has trained 581 participants. Participant outcomes include increased expertise in IPC and plans for improving or implementing IPC infrastructure at their institutions. IPC course attendees also communicate with each other as part of course assignments, and these virtual relationships continue after completion of the course, including through the Global ID networks.

#### Global health sessions of the annual St Jude/PIDS Pediatric Infectious Diseases Research Conference (St Jude/PIDS).

The global health session (GHS) is a program within the St Jude/Pediatric Infectious Diseases Society (PIDS)^13^ annual research conference conceived to satisfy a growing need for a dedicated forum in which participants could present and discuss IC&P developments that concern pediatric populations globally and to create opportunities for domestic and global participants to meet, learn from each other, and collaborate. The GHS, created by the Global ID program in 2017 and comanaged with the St Jude/PIDS conference, has the goal of exposing attendees to pediatric global health issues, stimulating participation in efforts to better understand health determinants that affect children around the globe and decrease IC&P health inequities. The GHS provides a setting for network members to present and discuss the results of their studies on infectious pathogens, especially those affecting children with depressed immunity, as poster or oral presentations.

#### The St Jude Global Alliance online community infectious disease portal.

The ID portal is a platform through which health care providers, including members of the Global ID networks, can connect, communicate, and collaborate. Through the ID portal, network members interact, ask questions, seek advice, and find information on topics of interest. The ID portal provides access to a collection of tools and educational resources, along with opportunities for project-specific interactions. Currently, this portal is password-protected, limiting access to members of the community.^11^

### Network Design and Development

#### Network design.

Graduates of the first ID course wished to continue communicating and cooperating with each other after the course ended in 2017.^10^ With that desire in mind, a professional network was thought to be a way to continue engagement and a network name was selected: PRINCIPAL, a Spanish acronym for Prevencionistas e Infectologos de Cancer Pediátrico en America Latina, meaning Preventionists and Infectologists for Pediatric Cancer in Latin America. Moreover, basic concepts, such as the vision, mission, and values, were established in discussion with course participants. After several iterations and corrections, the current vision, mission, and values were finalized and are provided in Table 2.

**TABLE 2:**
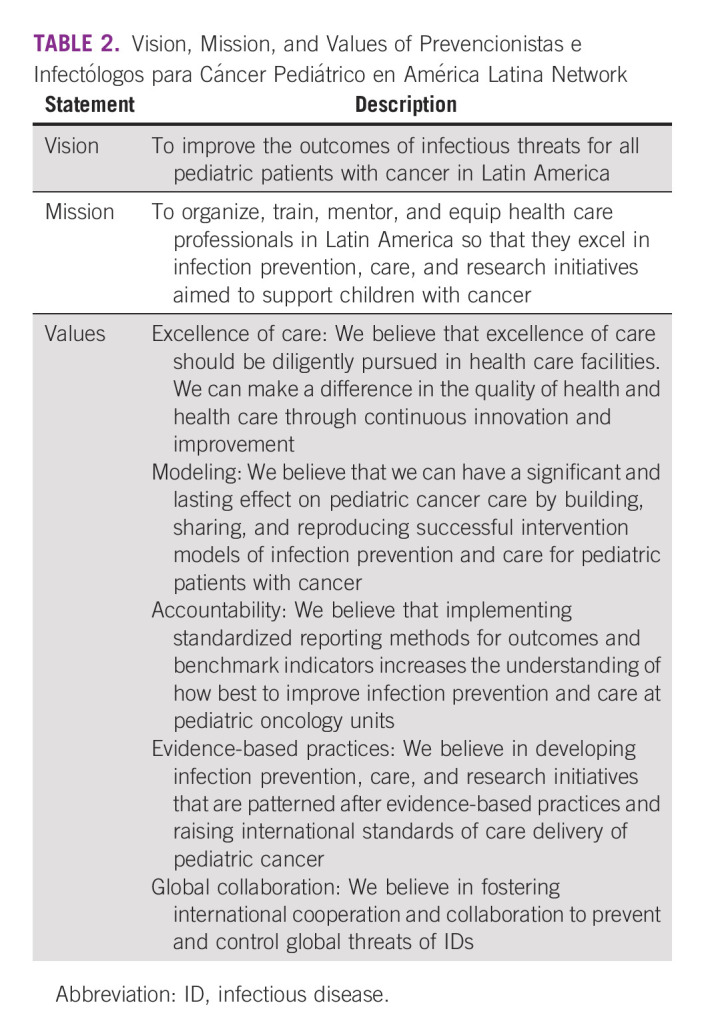
Vision, Mission, and Values of Prevencionistas e Infectólogos para Cáncer Pediátrico en América Latina Network

#### Network development.

Once the guiding concepts were formulated, we addressed issues such as membership eligibility and benefits, network activities, and communication venues, including internet messaging (WhatsApp). One early benefit of membership was the ability to obtain the support of fellow members as speakers at IC&P conferences and workshops to impart knowledge in IC&P in pediatric oncology. Requirements for membership included the following: employment or privileges at a health care facility that provides care to children with cancer or other catastrophic illnesses; involvement in IC&P activities; agreement to collaborate with Global ID and network members on strategic goals; and permission to engage in network activities from a direct work supervisor. The benefits of being a member now include access to additional education and training resources through Global ID and St Jude Global, opportunities to obtain support for travel to regional and international scientific meetings, opportunities to collaborate on multicenter research and collaborative studies, and the availability of support for study design and data management.

To engage network members and increase their knowledge of and expertise in IC&P in the areas of clinical care and research, we created the following resources:

- Case-based learning: We established a weekly event, *Kiosko de Casos*, to improve IC&P professional skills through the discussion of difficult and complex patient cases. These cases mainly involve patients with cancer or recipients of hematopoietic cell transplants.
- Research in human subject certification: We stimulated network members to become certified in human subject research by providing access to the Collaborative Institutional Training Initiative program.^14^ Collaborative Institutional Training Initiative training is mandatory for participating in network-associated research activities but is voluntary for other interested members.
- Quality improvement skills: We partnered with a Lean 6-Sigma instructor through the St Jude Department of Quality and Patient Safety to collaborate on and deliver a 6-month distance-learning training program through which participants earned Green Belt certification.^15^
- Cross-collaboration: The PRINCIPAL network has become a focal point for expertise in IC&P in immunocompromised children. As a result, network members have participated in multiple institutional, national, and regional IC&P meetings and conferences, collaborating with institutions and organizers of scientific meetings, short courses, and seminars as speakers on identified gaps in IC&P topics in immunocompromised patients^16^ (Table 3).
- Presentation of professional work: We have encouraged members to share local experiences, the results of clinical research, and the outcomes of quality improvement efforts in IC&P at scientific conferences, particularly at the St Jude/PIDS GHS (Table 4).
- Participating in research related to IC&P: We have also encouraged network members to establish and/or collaborate in clinical or basic research and qualitative studies.^16-21^
- Network annual meeting: Since the network was formally established in 2017, an annual meeting has been scheduled every year thereafter. The meeting was held in person before the pandemic (2018-2019) and was virtual in 2020-2022. In these meetings, we review the state of the network and provide reports on achievements and future directions.
- Monthly bulletin: We produce a monthly bulletin in which we announce upcoming events and summarize the results of ongoing activities. Every other month, we invite network members to contribute a review of a recently published IC&P paper, to share their experiences in IC&P, and/or to communicate some important facts regarding IC&P. This bulletin is distributed to all graduates of our courses and other interested individuals. After publication, the issues are archived and are accessible through our ID portal.

**TABLE 3:**
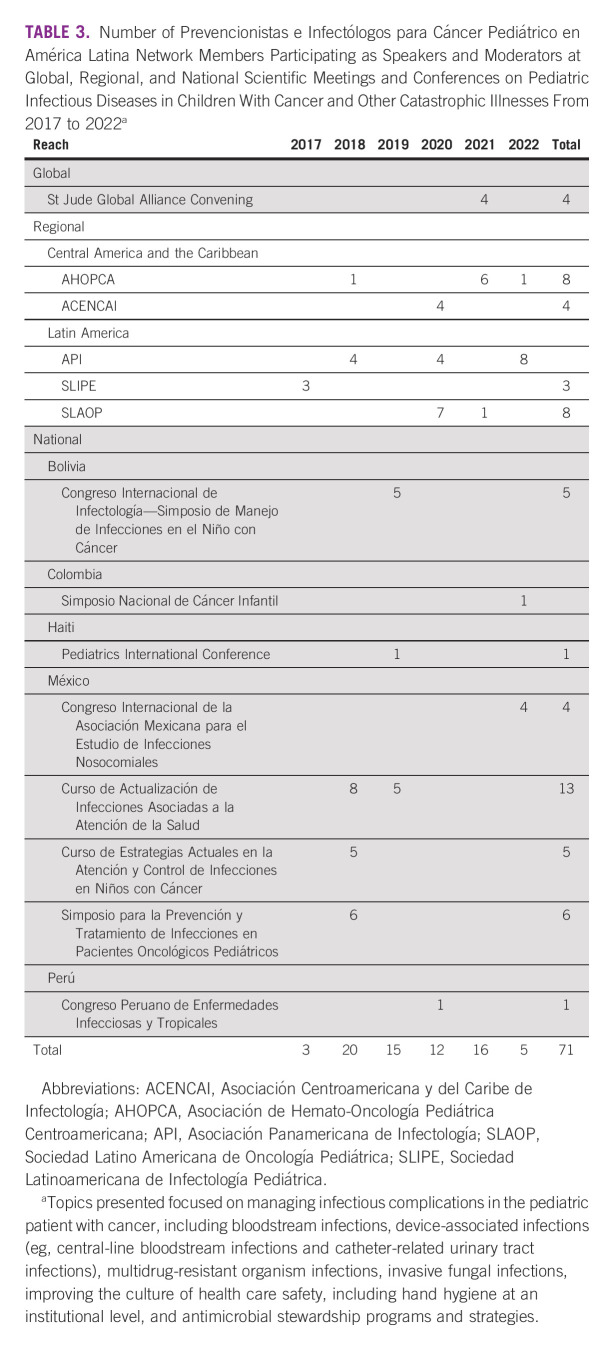
Number of Prevencionistas e Infectólogos para Cáncer Pediátrico en América Latina Network Members Participating as Speakers and Moderators at Global, Regional, and National Scientific Meetings and Conferences on Pediatric Infectious Diseases in Children With Cancer and Other Catastrophic Illnesses From 2017 to 2022^a^

**TABLE 4:**
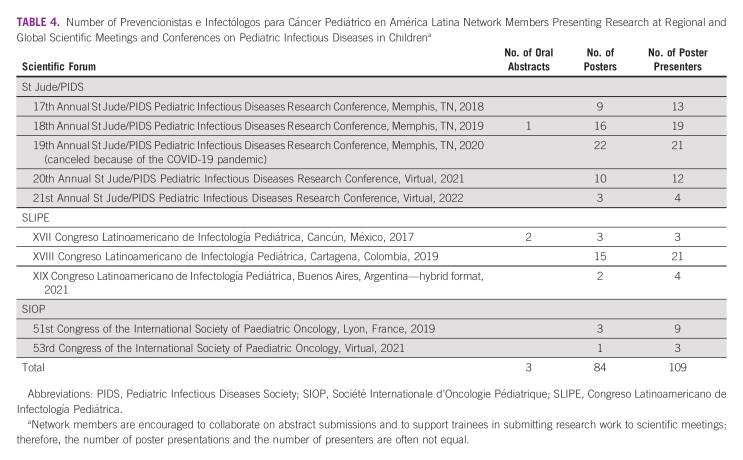
Number of Prevencionistas e Infectólogos para Cáncer Pediátrico en América Latina Network Members Presenting Research at Regional and Global Scientific Meetings and Conferences on Pediatric Infectious Diseases in Children^a^

#### PRINCIPAL network structure and leadership.

The PRINCIPAL network activities are coordinated by the Global ID program with the input of members. This includes enrolling new members, coordinating meetings, finding speakers, and assisting working groups (WGs) in executing their projects. To integrate the PRINCIPAL network into a global structure, representatives of this network serve on the recently formed Global ID network steering committee (SC). This committee will provide a leadership structure that will in turn lead to a more sustainable and participatory organization. Committee members are currently working to outline the duties of the committee, as well as the roles of the WGs. Leadership consists of network sponsors, SC members, and WG leaders. The WGs will execute projects as they emerge, on the basis of the interest and resources available. Support of these WGs will be essential to ensure productivity across the domains of education, capacity building, and research.

##### Members.

Prospective members of the network must be qualified by their training, be involved in pediatric oncology care, and have the support of their workplace. Network membership is free of charge but requires completing an application and periodic renewal. The benefits of membership include invitations to all network-related activities and initiatives, as well as leadership participation. Members also have the opportunity to apply for travel awards for attending scientific conferences and courses, and they receive assistance with finding support for project execution and publication.

##### Sponsors.

The sponsors speak about and advocate for the network. They promote and discuss its function and accomplishments, and they assist with matters relating to representation, the procurement of resources, and funding. Currently, the Global ID program is the main sponsor of the network.

##### Funding.

Department and programmatic resources support network operations and maintenance. Educational programs and conference awards are funded through the Global ID budget, but members are encouraged to apply for conference-sponsored awards as well as other funding opportunities circulated by the network.

##### Global ID network SC.

The SC deliberates, makes decisions, advises, provides strategic oversight, and serves as the primary advocate for initiatives carried out by WGs. Their roles are to build, review, and amend (if needed) long- and short-term goals, objectives, and strategies; to guide network activities on the basis of these goals, objectives, and strategies; to monitor network activities; and to develop ideas to advance network objectives by conducting periodic evaluations of the network. The members of the inaugural SC were selected by the sponsors, and subsequent members will be selected by the departing SC members and the sponsors. SC will facilitate the transition of network activity ownership to its members. This will include, but will not be limited to, leading and managing network project initiatives and organizing coordination and administrative support.

##### Working groups.

The performance of the network will be reflected in the participation, productivity, and diverse themes of the WGs, categorized into three groups: education, capacity building, and research. These WGs, which are highly collaborative, will be led by interested members and may be composed of fellow network members or nonmembers. Before activation, the WG proposal is reviewed and approved by the SC. To date, there are three operational WGs in the global network and one WG exclusively for the PRINCIPAL network region (the use of a clinical care pathway in the initial management of a pediatric patient with fever).

## RESULTS

### Network outcomes.

Since inception, network productivity has been reflected in the number of members participating in various activities in the areas of education, collaboration, advocacy, and research. Importantly, network members have shared their work at meetings through abstracts and poster/oral presentations (Tables 3 and 4) and in selected publications (Table 5). We anticipate that the lasting effect of the network will be an improvement in the use of evidence-based practice and data standardization. Report standardization begins by teaching disease definitions and their use in our courses,^10,12^ which can be followed by mentoring IC&P teams at local sites.^23^ Recently, we concluded a 3-year mentoring project of IC&P teams, members of the PRINCIPAL network, in three hospitals on Hispaniola Island, two in the Dominican Republic, and one in Haiti.^24-26^ A PRINCIPAL network member also concluded the experience in standardizing central line–associated bloodstream infections in another institution in Latin America.^27^ The ID portal provides resources and training on disease definitions as well.^11^ Building these resources for data management and interpretation is ongoing.

**TABLE 5:**
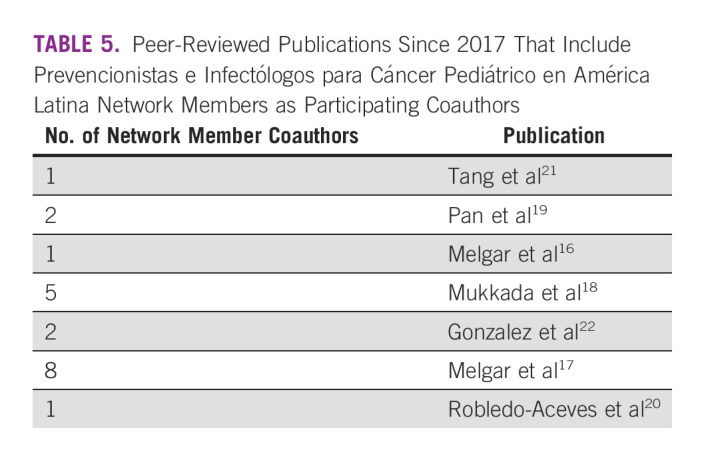
Peer-Reviewed Publications Since 2017 That Include Prevencionistas e Infectólogos para Cáncer Pediátrico en América Latina Network Members as Participating Coauthors

### Network challenges and barriers.

The emergence and resolution of the multiple challenges and barriers during the life of the network have been dynamic. These were finding a model for building a network such as PRINCIPAL; developing suitable and attractive activities for members; developing performance evaluation indicators (process and outcomes affecting patient care); building and maintaining a framework to respond to membership demands; defining leadership structure and roles and responsibilities of members and administrative personnel; and developing a plan to sustain the network. These shortcomings were addressed by consulting with experts, using helpful ideas from published literature, collaborators, and importantly from members of the network.

### Future Directions and Sustainability

We expect the PRINCIPAL network to be a venue for building and bringing expertise to pediatric oncology centers throughout Latin America. The PRINCIPAL network must continue to grow and support local and regional expertise in IC&P in pediatric cancer. The members are a critical resource for the clinical workforce, educators, and investigators in IC&P in pediatric cancer. Importantly, through the network, members can unify their voices, message, and efforts at the institutional, country, and regional level and bring their collective thoughts as a network to the global forum. To capitalize on the enthusiasm of new members, robust support for education, capacity building, and research must be in place. There is also a clear need for the network to provide mentoring to WGs. To meet this need, the SC and WG members are identifying partnerships with other networks, professional organizations, and higher education institutions that will result in mutual benefits. PRINCIPAL network members have begun this type of engagement with the International Society for Pediatric Oncology (Société Internationale d'Oncologie Pédiatrique) Supportive Care and the Société Internationale d'Oncologie Pédiatrique Global Health networks in IC&P in pediatric oncology. These networks are excellent sources of expertise in patient care and collaborators in IC&P. The PRINCIPAL network is evolving as a model for establishing collaboration, data sharing, resource procurement, and IC&P proficiency globally.

## DISCUSSION

In a collaborative undertaking, we designed and operationalized a regional network for professionals involved in IC&P in Latin America. This network, initially in response to the desire of initial members to continue engaging with each other after finishing a training course, is becoming a model for the global engagement of professionals in sharing ideas and opportunities for collaboration in IC&P and serves as a model for creating other regional networks. Born of necessity, assembling the network incorporated the essentials, namely the mission, vision, and values, and the rules for engaging stakeholders. Well-functioning networks can speed communication, grow collaboration, and rapidly introduce changes, providing benefits unavailable to individuals working alone. These qualities are needed to confront the ever-growing threats to global health, including the ongoing COVID-19 pandemic.

Other professional networks have been created in response to a natural disaster^28^ or to study a disease common in children.^29,30^ As with our network, the structure of these networks became more defined over time. The participation of members in identifying the optimal structure and continuously striving to ensure the network satisfies the growing needs of its members can guide not only its inauguration but also measures to sustain it.^31^

The PRINCIPAL network and its members aim to improve the outcomes of infectious threats in the pediatric oncology population at engaged institutions in Latin America. In REKAMLATINA (Red de Enfermedad de Kawasaki en América Latina), members identified challenges in dealing with Kawasaki disease in Latin America and suggested actions to improve outcomes.^29,30^ Health care professions such as nursing see structured gatherings as important ways to stimulate conversations, learn about emerging ideas, and find support for the needs of members.^32^ The Federation of Gynecology and Obstetrics, a global advocate for the health of women and children, originated as a structure to address the pressing need for measures to prevent maternal and newborn deaths in Africa and Southeast Asia.^33^ However, as with our network, the engagement of members continued and expanded, and the Federation of Gynecology and Obstetrics is currently the only organization that brings together professional societies of obstetricians and gynecologists on a global basis.^34^ Guided by its foundational mission, vision, and values, and through its inclusive leadership structure, the PRINCIPAL network will engage its members and sponsors, focusing specifically, but not exclusively, on pediatric cancer regionally and globally. Building expertise by participating in IC&P for patients with a rare disease such as cancer can take a long time. Furthermore, the variety and complexity of pediatric cancers and the rapid evolution of new therapies and emerging complications necessitate continuous learning. By using its resources, our network will enable its members to share their experience and knowledge of IC&P in pediatric cancer and will speed the learning process, thereby developing local expertise necessary for better IC&P outcomes in a given region.

Implementing the PRINCIPAL network included challenges, but sustaining it imposes additional factors to overcome. A survey of the specialty of Pediatric Infectious Diseases in Latin America found that scarcity of support personnel, time availability, outside-the-specialty medical duties at multiple institutions, high patient volume, and lack of protective time for research and administrative duties were limiting factors for pediatric ID physicians' participation in academic endeavors including those promoted by the PRINCIPAL network.^35^ Furthermore, the infrastructure for research and data collection is often suboptimal at many participating institutions, and ethics committees may not be familiar with the types of research conducted by network members. Therefore, participation in WGs often requires effort characterized by excellent time management, temperate character, and high motivation.

The benefits of participating in the PRINCIPAL network are quickly emerging. Outcomes amenable to measurement include the number of members participating in conferences, research collaborations, and publications. The intangible benefits for members are far more abundant and difficult to measure, but they are no less important. They include developing new skills and competencies, sharing expertise and knowledge, linking up with colleagues, and contributing to regional proficiency and resources. According to Mata et al,^36^ the values of belonging to a professional organization are numerous. These benefits are especially useful for young professionals, for whom network activities can provide a place for interaction, sharing, and finding collaborators and mentors, especially in areas of common interest. Another important benefit of the network is the ability to gain access to members from all areas of a vast region such as the Americas for collaborative studies, especially ones aimed at learning the regional status of health or health care. It is frequently recommended that individuals initiating academic work, especially in health sciences, network for collaborations.^37^ Despite the intricacies of some collaborative arrangements, learning how to navigate them is crucial for healthy growth of the network.^38,39^ Recognizing the needs of network members and their role in education, capacity building, and research as part of building the network, the sponsors offered to provide additional assistance with respect to improving clinical skills, thematic training sessions, quality improvement, and leadership.

Building a professional network has stimulated knowledge exchange, cross-mentoring, and collaboration among our course graduates. Strengthening the network structure and operations, recruiting course graduates as members, and retaining them in the network have emerged as essential to the long-term objectives of our course. Following the course, we assessed members' confidence with clinical care, collaborations, and academic productivity in IC&P,^10^ and continue to monitor these outcomes. To optimize the use of limited resources and to ensure the success of health care interventions, health care providers in pediatric cancer centers in LMICs, being themselves limited in number,^40^ need to be master clinicians and need to know about the best practices in IC&P.^35^ Therefore, improving the knowledge of IC&P among health care providers is the first step in building an effective institutional workforce and sustaining the quality of health care for pediatric patients with cancer. We expect that better expertise will promote better patient care and better quality of educational content and that it will stimulate studies about local issues and areas of interest. Considering that, in any country, the number of professionals with capabilities in IC&P for pediatric cancer are concentrated in a few tertiary care hospitals, the effect of improving the quality of IC&P professionals can be considerable. A similar effect can occur with training to promote research leadership skills. Other focused specialties, such as pediatric critical care, stress the importance of partnerships and networking to promote the global specialty agenda.^41^ The PRINCIPAL network provides members with a suitable environment in which to communicate their research and, importantly, to identify best practices and opportunities for improvement. As network members, they can collaborate widely with members from other regions, uniting their voices to present their concerns to global agencies to advocate for greater commitment for improving care.

The resources provided by network member contributions include building and teaching in trainings, speaking at conferences, providing expertise for difficult IC&P cases, and collaborating research initiatives. Our training courses and the resulting community of graduates operating in networks can build and sustain IC&P knowledge and expertise globally. Participating in our IC&P networks will augment attendees' professional contributions locally, stimulate their leadership potential, and encourage their collaboration in professional networks. Ongoing efforts continue to target IC&P training, thereby strengthening local human capacity and augmenting local and collective expertise, which results in improved care and prevention of infection in children with cancer.

In conclusion, the St Jude Global ID program built a regional network, PRINCIPAL, to engage members, build their expertise, disseminate new IC&P evidence-based information on best practices, and collaborate in clinical, quality improvement, and implementation research. A professional network for health care professionals who are interested in and dedicated to improving IC&P will rapidly enhance the impact of educational, capacity building, and research interventions in a region. The PRINCIPAL network is a model for building a similar network in any geographic region that aims to rapidly deploy expertise building and collaboration.
